# MiR-101 and miR-144 Regulate the Expression of the CFTR Chloride Channel in the Lung

**DOI:** 10.1371/journal.pone.0050837

**Published:** 2012-11-30

**Authors:** Fatemat Hassan, Gerard J. Nuovo, Melissa Crawford, Prosper N. Boyaka, Stephen Kirkby, Serge P. Nana-Sinkam, Estelle Cormet-Boyaka

**Affiliations:** 1 Department of Internal Medicine, Division of Pulmonary, Allergy, Critical Care and Sleep Medicine, The Ohio State University, Columbus, Ohio, United States of America; 2 The Ohio State Comprehensive Cancer Center, The Ohio State University, Columbus, Ohio, United States of America; 3 Department of Veterinary Biosciences, The Ohio State University, Columbus, Ohio, United States of America; Abramson Research Center, United States of America

## Abstract

The Cystic Fibrosis Transmembrane conductance Regulator (CFTR) is a chloride channel that plays a critical role in the lung by maintaining fluid homeostasis. Absence or malfunction of CFTR leads to Cystic Fibrosis, a disease characterized by chronic infection and inflammation. We recently reported that air pollutants such as cigarette smoke and cadmium negatively regulate the expression of CFTR by affecting several steps in the biogenesis of CFTR protein. MicroRNAs (miRNAs) have recently received a great deal of attention as both biomarkers and therapeutics due to their ability to regulate multiple genes. Here, we show that cigarette smoke and cadmium up-regulate the expression of two miRNAs (miR-101 and miR-144) that are predicted to target CFTR in human bronchial epithelial cells. When premature miR-101 and miR-144 were transfected in human airway epithelial cells, they directly targeted the CFTR 3′UTR and suppressed the expression of the CFTR protein. Since miR-101 was highly up-regulated by cigarette smoke *in vitro*, we investigated whether such increase also occurred *in vivo*. Mice exposed to cigarette smoke for 4 weeks demonstrated an up-regulation of miR-101 and suppression of CFTR protein in their lungs. Finally, we show that miR-101 is highly expressed in lung samples from patients with severe chronic obstructive pulmonary disease (COPD) when compared to control patients. Taken together, these results suggest that chronic cigarette smoking up-regulates miR-101 and that this miRNA could contribute to suppression of CFTR in the lungs of COPD patients.

## Introduction

CFTR is a chloride channel that is primarily expressed at the apical surface of airway epithelial cells and is involved in the control of airway surface fluid homeostasis [1]. Absence of functional CFTR is known to cause Cystic Fibrosis with lung-related problems being the leading cause of mortality [2]. CFTR expression can be regulated at the transcriptional and post-transcriptional levels. CFTR interacts with many proteins that can affect its stability, degradation, and/or processing [3]. On the other hand, few copies of CFTR mRNA have been found in airway epithelial cells [4] suggesting that translational repression and/or mRNA degradation would strongly impact the amount of CFTR protein.

MicroRNAs (miRNAs) are short non-coding RNAs of about 22 nucleotides [5], which mainly function by translational repression and/or mRNA degradation by binding to the 3′ Untranslated Region (UTR). Therefore, down-regulation of miRNAs will result in increased protein expression of the targeted gene(s) whereas up-regulation of miRNAs will lead to suppression of the targeted protein(s). Deregulation of miRNAs has been found in many diseases including lung cancer and chronic obstructive pulmonary disease (COPD) [6], [7]. Up to 30% of human protein coding genes may be regulated by miRNAs [8]. Some pathological conditions lead to the loss of certain miRNAs such as Let-7 members in cancer. A single miRNA can target several mRNAs and multiple miRNAs can target the same gene. It was only recently that CFTR was found to be regulated by miRNAs [9], [10].

In this study, we investigated the effect of airway pollutants (cigarette smoke and cadmium) on miRNAs predicted to target CFTR *in vitro* in human airway epithelial cells as well as *in vivo* in the lung of smoke exposed mice and COPD patients. We also determined the role of miR-101 and miR-144 in regulating CFTR expression.

## Materials and Methods

### Ethics Statement

This study was carried out in strict accordance with the recommendations in the Guide for the Care and Use of Laboratory Animals of the National Institutes of Health. The protocol was approved by the Institutional Laboratory Animal Care and Use Committee (ILACUC) of the Ohio State University (protocol number: 2007A0168-R1).

### Tissue Culture and Reagents

The human bronchial epithelial cell line 16HBE14o- (HBE), a gift from Dr. Gruenert [11], was cultured in Dulbecco’s modified Eagle’s medium (DMEM) containing L-glutamine, 10% FBS and penicillin (100 U/ml) and streptomycin (100 µg/ml). The tissue culture plates were coated using human fibronectin (1 mg/ml), collagen I bovine (3 mg/ml), and bovine serum albumin (1 mg/ml). HEK-293 cells were cultured in DMEM containing 10% bovine growth serum, penicillin and streptomycin. Cell cultures were grown and maintained at 37°C in a 5% CO_2_ humidified incubator.

### Subjects and Sample Collection

Human lung samples were obtained from the Lung Tissue Research Consortium (LTRC, NIH) approved project (Concept Sheet #09-99-0017). The LTRC Patients were classified into two groups based on lung function tests with GOLD 4 having an FEV_1_/FVC <70%, FEV_1_<30% predicted or <50% normal with chronic respiratory failure, and GOLD 0 being asymptomatic with normal lung function.

### Animals and Lung Tissue Processing

C57BL/6 female mice (8–10 weeks old) were used in accordance with the institutional animal welfare guidelines of the Ohio State University. Mice were subjected to smoke from 3 standard cigarettes/day (Camel brand), 5 days/week for 4 weeks using a Teague 10 smoke machine. This is approximately the equivalent of 60–90 minutes of smoke exposure per day. Mice were sacrificed and lungs were inflated for histology.

### Cell Transfection and Immunoblotting

HBE cells were transfected with premiR-101, premiR-144 or a scrambled premiR control (Ambion, Austin, Texas) using Lipofectamine 2000 reagent (Invitrogen) according to the manufacturer’s instructions. Forty eight hours after transfection, HBE cells were lysed in PBS-1% Triton X-100 containing a cocktail of protease inhibitors (Roche Diagnostics, IN) for protein analysis. For mature miRNA analysis, total RNA was extracted using Trizol® (Invitrogen) as previously described [12]. CFTR protein was analyzed by immunoblotting after transfer to PVDF membranes (Bio-Rad, Hercules, CA). Primary CFTR antibody (24-1, R&D Systems) and β-actin (Santa Cruz Biotechnology) were added at a dilution of 1∶2,000 and 1∶10,000, respectively. HRP-conjugated secondary antibody (Pierce, Rockford, IL, USA) was used at 1∶10,000. The signal was detected using West Pico (Pierce, Rockford, IL).

### Luciferase Assay

The 3′UTR (untranslated region) of CFTR was amplified by RT-PCR out of genomic DNA. The amplified products were sub-cloned into psiCHECK-2 vector (Promega, Madison, WI). In addition, we conducted mutagenesis of the seed sequence present in the 3′UTR to prevent binding of the specific miRNAs. The mutations were confirmed by sequencing. HEK-293 cells were transfected with 50 ng of psiCHECK-CFTR or psiCHECK empty vector and either scrambled pre-miR, pre-miR-101, or pre-miR-144. Twenty four hours later, cells were assayed for both firefly and renilla luciferase using the dual luciferase glow assay (Promega, Madison, WI) and Victor™ X3 fluorescent plate reader (PerkinElmer, MA).

### Quantitative RT-PCR (qRT-PCR) Analysis

Real-time quantitative RT-PCR was employed to measure the transcript levels of mature miR-101 and miR-144. RT-PCR was performed using TaqMan microRNA Reverse Transcription kit (Applied Biosystems, CA) following manufacturer’s protocol and assayed on the Applied Biosystems 7900HT. The primers for miR-101, miR-144, and miR-145 were purchased from Applied Biosystems and U6 snRNA was used as endogenous control. Data are expressed as relative copy number (RCN), which was calculated using with the following equation: RCN = 2^–ΔCt^ x100 where ΔCt = Ct_(target)_–Ct_(housekeeping gene)_ [13].

### In situ Hybridization

Detection of miRNAs in paraffin-embedded tissues was performed as previously described [14]. The locked nucleic acid (LNA) modified cDNA probe complementary to human mature miR-101 was used (Exiqon Inc, MA). The probes were labeled at the 5′ end with digoxigenin by the manufacturer. The negative controls include omission of the probe and the use of a scrambled probe.

### Immunohistochemistry

Our immunohistochemistry protocol has been previously published [15]. In brief, the antibodies were optimized by comparing no pretreatment, protease digestion, antigen retrieval, or antigen retrieval plus protease digestion using positive control tissues with the automated Benchmark LT platform (Ventana Medical Systems). The detection systems used were the Ultraview Fast Red and the Ultraview DAB based systems; hematoxylin served as the counterstain with each detection system. The optimal conditions for CFTR detection by immunohistochemistry was a dilution of monoclonal CFTR antibody (R&D Systems) at 1∶200 with antigen retrieval for 30 minutes prior to immunohistochemistry.

### Statistical Analysis

Data are expressed as mean ± standard errors (SE) of at least three independent experiments. Statistically significant differences were assessed using Student’s t-test. P values <0.05 were considered significant.

## Results

### Cigarette Smoke and Cadmium Induce Up-regulation of miR-101 and miR-144

**Figure 1:**
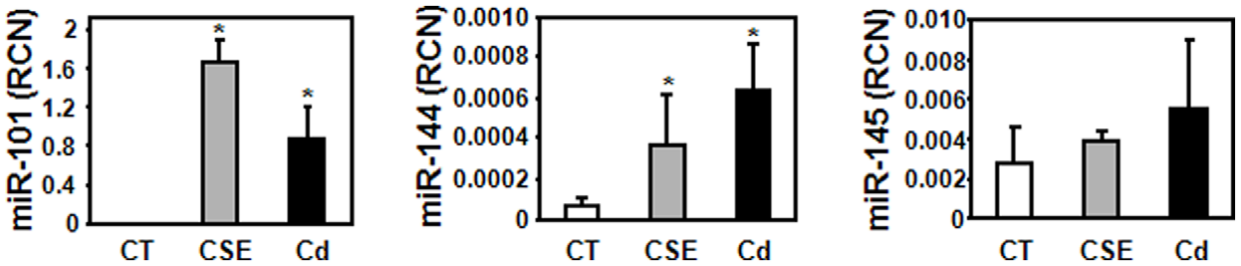
Effect of the air pollutants cigarette smoke and cadmium on expression of miR-101, miR-144, and miR-145. Human bronchial epithelial cells (HBE) were treated with 5% cigarette smoke extract (CSE) or 2 µM cadmium (Cd) for 24 hours. Total RNA was isolated and expression of mature miR-101, miR-144, and miR-145 was measured by quantitative RT-PCR. Data are representative of at least three independent experiments. *p<0.05.

We previously showed that the air pollutants cigarette smoke and cadmium suppress the expression of the CFTR chloride channel in human airway epithelial cells [13], [16]. We therefore exposed human bronchial epithelial (HBE) cells to cigarette smoke extract and cadmium for 24 hours. The expression of three miRNAs predicted to target CFTR (miR-101, miR-144, and miR-145) was determined. Exposure of HBE cells to cigarette smoke resulted in ≈80- and 4-fold increases of miR-101 and miR-144, respectively, while cadmium induced miR-101 and miR-144 by ≈40 and 6 fold (Fig. 1). Conversely, neither cigarette smoke extract nor cadmium increased the expression of miR-145 (Fig. 1).

### Expression of miR-144 and miR-101 Suppresses CFTR Protein in HBE Cells

**Figure 2:**
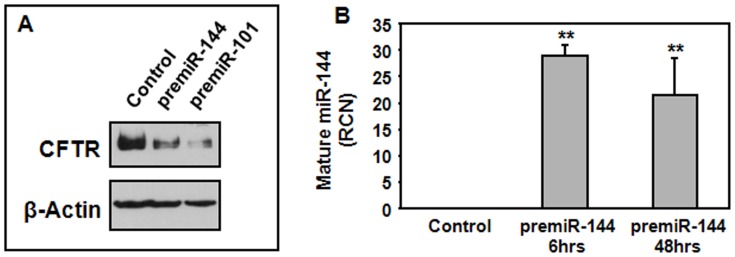
Expression of miR-101 and miR-144 decreases expression of CFTR protein. HBE cells were transfected with 30 nM of pre-miR-101 or pre-miR-144 using Lipofectamine 2000. (A) Expression of CFTR was detected 3 days post-transfection. (B) Expression of mature miR-144 was measured by quantitative RT-PCR 6 and 48 hours after transfection. **p<0.001.

Since miR-101 and miR-144 are predicted to target the CFTR gene, we evaluated the effect of these miRNAs on the expression of CFTR protein. We therefore transfected each miRNA as a precursor (premiR) in HBE cells that constitutively express the CFTR protein. Transfection with premiR-101 or premiR-144 resulted in suppression of the CFTR protein as observed in Figure 2A. The expression of mature miR-101 and miR-144 was confirmed by quantitative RT-PCR. Mature miR-101 and miR-144 could be detected six hours post-transfection and were still highly expressed 48 hours after transfection (Fig. 2B and data not shown).

### MiR-101 and miR-144 Target CFTR 3′UTR

**Figure 3:**
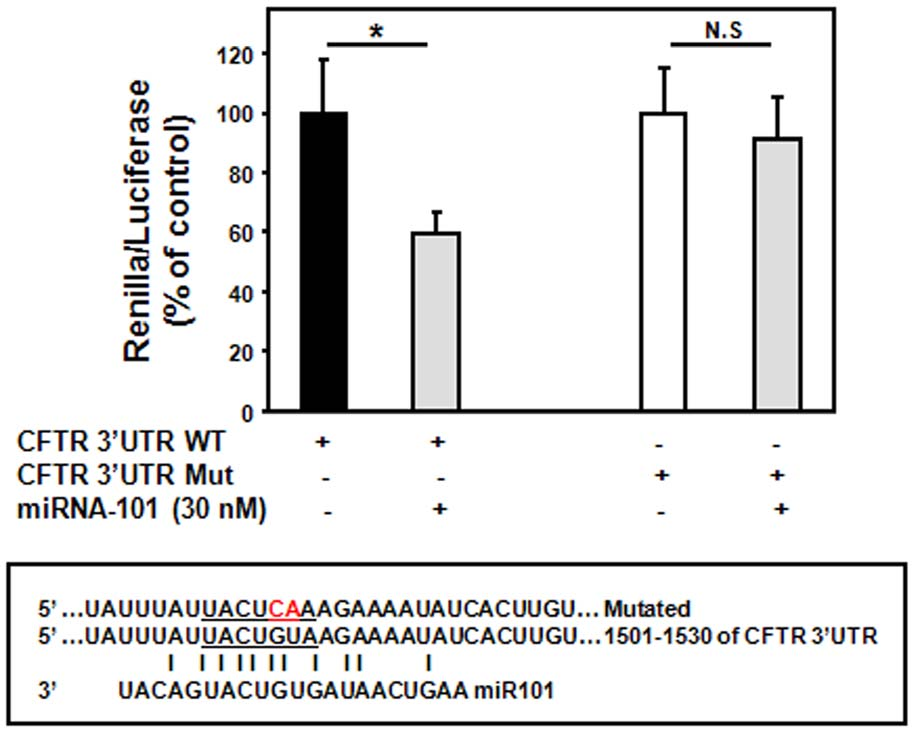
MiR-101 targets 3′UTR of CFTR. Cells were transfected with 50 ng of psiCHECK containing wild-type (WT) or mutated (Mut) CFTR 3′UTR and 30 nM of pre-miR-101. Twenty four hours following transfection, cells were assayed for both firefly and renilla luciferase using the dual luciferase glow assay. All transfection experiments were conducted in triplicate. Data are expressed as mean±SE of at least three independent experiments. *p<0.05, n.s.: not significant.

**Figure 4:**
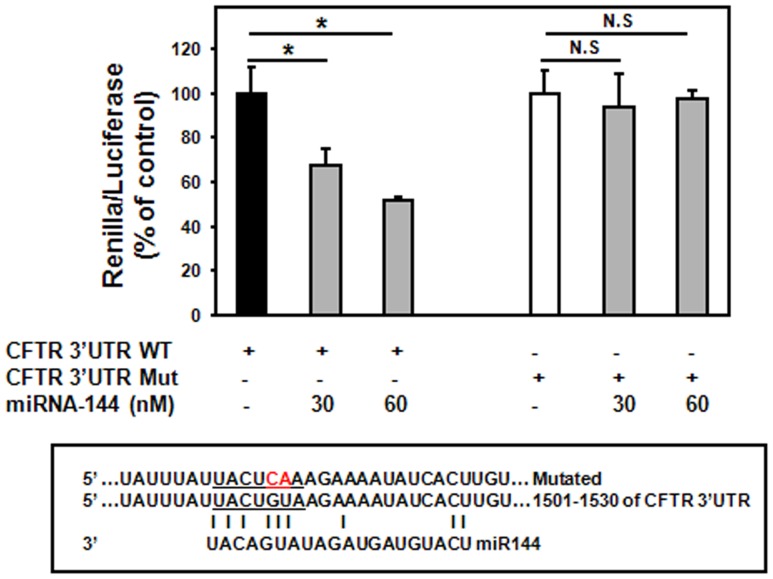
MiR-144 targets 3′UTR of CFTR. Cells were transfected with 50 ng of psiCHECK containing WT or Mut CFTR 3′UTR and either 30 or 60 nM of pre-miR-144. Twenty four hours following transfection, cells were assayed for both firefly and renilla luciferase using the dual luciferase glow assay. All transfection experiments were conducted in triplicate. Data are expressed as mean±SE of at least three independent experiments. *p<0.05, n.s.: not significant.

In order to confirm that miR-101 and miR-144 directly target CFTR, the CFTR 3′UTR was subcloned into the reporter psiCHECK-2 vector. As indicated in Figure 3, expression of miR-101 reduced the reporter activity by ≈40%. Similarly, overexpression of miR-144 resulted in ≈30 and 50% decrease in reporter activity when cells were transfected with 30 and 60 nM of pre-miR-144, respectively (Fig. 4). In order to validate the binding specificity, the seed sequence for CFTR 3′UTR was mutated (Figs. 3 and 4). Mutations in the CFTR 3′UTR (CFTR 3′UTR Mut: GU to CA) eliminated the effect of miR-101 and -144 on reporter activity (Figs. 3 and 4).

### MiR-101 is Overexpressed in the Lung of Mice Subjected to Cigarette Smoke

**Figure 5:**
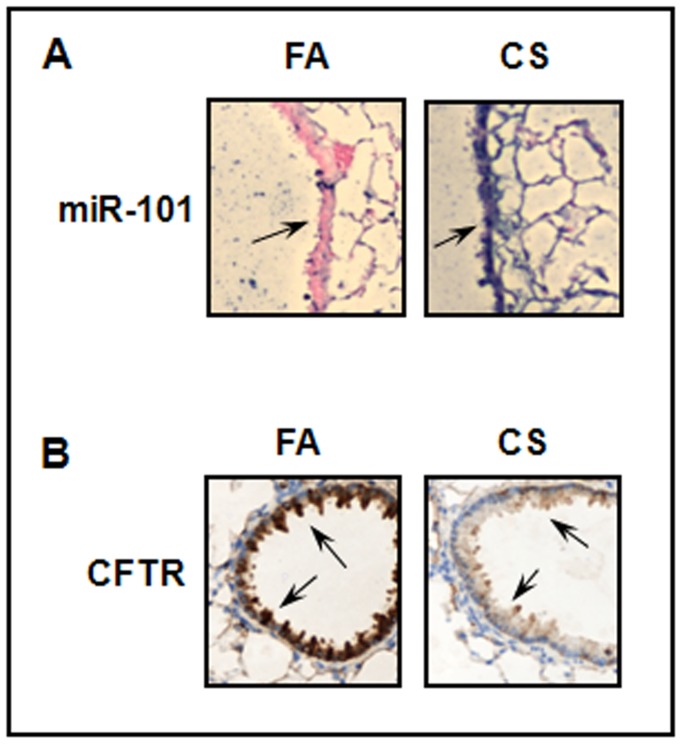
Detection of miR-101 in the lung of mice subjected to cigarette smoke. Mice were subjected to filtered air (FA) or cigarette smoke (CS) for 4 weeks. (A) Paraffin-embedded, formalin-fixed lung tissues were incubated with an LNA probe anti-miR-101 (purple staining), or scrambled probe as previously described [12]. (B) CFTR protein (brown staining) was detected by immunohistochemistry as described in methods section. Arrows show the bronchial epithelium. The images are representative of 3–4 mice for each condition.

Taken together, our results above show that air pollutants (such as cigarette smoke and cadmium) induce up-regulation of miRNAs that target CFTR resulting in suppression of CFTR protein in airway epithelial cells *in vitro*. In order to determine whether such phenomenon can be observed *in vivo*, mice were subjected to cigarette smoke for four weeks. We focused on miR-101 since this miRNA was the most highly up-regulated by cigarette smoke *in vitro*. MiR-101 (purple staining) was found to be highly expressed in bronchial epithelial cells and in alveolar cells in the lung of mice subjected to cigarette smoke when compared to mice exposed to filtered air (Fig. 5A). Since we previously showed that miR-101 targets CFTR, we next investigated the expression of the CFTR protein. We found that CFTR protein (brown staining) was greatly reduced in the lung of mice subjected to cigarette smoke as observed by immunohistochemistry and more specifically in bronchial epithelial cells (Fig. 5B).

### MiR-101 is Highly Expressed in the Lung of COPD Patients

**Figure 6:**
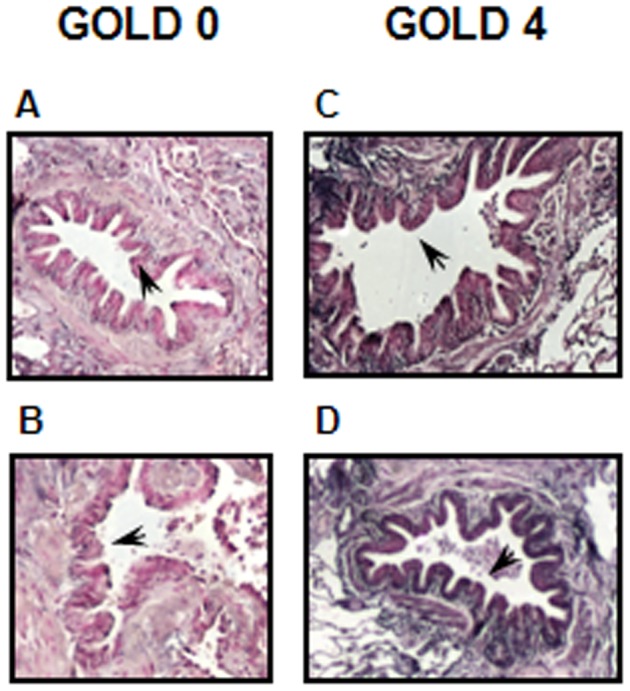
Detection of miR-101 in the lung of control (GOLD 0) and GOLD 4 COPD patients. Paraffin-embedded, formalin-fixed lung tissues from control (GOLD 0) (A&B) or patients with severe COPD (GOLD 4) (C&D) were incubated with an LNA probe anti-miR-101 (purple staining). The bronchial epithelium is shown by arrows. Images are representative of four patients per group.

We recently reported that CFTR protein was suppressed in the lung of COPD patients with a history of smoking [16]. Therefore, we investigated whether miR-101 was upregulated in the lung of these COPD patients. As observed in Figure 6, miR-101 (purple staining) was strongly expressed in bronchial epithelial cells in patients with severe COPD (GOLD 4) when compared to control patients (GOLD 0).

## Discussion

CFTR is a chloride channel that plays a critical role in maintaining fluid homeostasis in the lung. Thus, mutations in the *cftr* gene that result in absence or malfunction of the CFTR protein lead to cystic fibrosis, a disease characterized by impaired mucus clearance, chronic infection and inflammation. We recently reported that air pollutants, such as cigarette smoke and cadmium, reduce the expression of the CFTR protein in vitro in airway epithelial cells [13]. Here we show that cigarette smoke and cadmium induce up-regulation of two miRNAs that target CFTR, reducing its expression in human airway epithelial cells. We further show that miR-101 is up-regulated in the lung of mice subjected to cigarette smoke and in COPD patients.

There is increasing evidence that airway pollutants such as cigarette smoke suppress the expression of the CFTR protein [17], [18]. We and Bodas et al., recently showed that CFTR is suppressed in the lung of COPD patients suggesting that reduced expression of CFTR could contribute to the development of this disease [16], [19]. Here we show that cigarette smoke and the toxic metal cadmium induce up-regulation of specific miRNAs that target CFTR. Gillen et al. recently reported that CFTR can be regulated by several miRNAs including miRNA-144 but did not observe any effect of miR-101 on CFTR [10]. The discrepancy in the results could be due to the model used; human colon cancer cells versus human bronchial epithelial cells. It is therefore possible that expression and regulation of miRNA-101 is cell-type specific but also depends on the disease state (normal or cancerous). Interestingly, miR-101 was reported to play a role in inflammation by targeting MAPK phosphatase-1 (MKP-1), a dual specific phosphatase that deactivates MAPKs, which functions as a negative regulator of the innate immune system [20], [21]. We can speculate that high expression of miR-101 observed in the lung samples could contribute to the sustained activation of Erk1/2 (phosphoErk1/2) observed in COPD patients [22] due to lack of dephosphorylation by MKP-1.

Regarding miR-144, this miRNA has been found to be elevated in cancer [23]-[25], and was recently identified to be among the top three miRNAs up-regulated in the lung of COPD patients [7]. MiR-101 and miR-144 target the same region of CFTR 3′UTR and share the same seed sequence indicating that these two miRNAs do not act synergistically or additionally. On the other hand, the fact that both miR-101 and miR-144 target the same region suggests that this 3′UTR region is highly regulated by miRNAs.

Cigarette smoke and cadmium similarly affected two of the three miRNAs investigated in this study, all predicted to target CFTR. Both pollutants increased miR-101 and miR-144 but had no effect on miR-145. Since cadmium is a contaminant of cigarette smoke, it is possible that cadmium present in cigarette smoke was responsible for the up-regulation of miR-101 and miR-144. Interestingly, the cytokine IL-17A was recently identified to up-regulate miR-101 via activation of the Akt pathway in cardiac fibroblasts [21]. Since both cigarette smoke and cadmium activate the Akt pathway [26]-[28], it is possible that up-regulation of miR-101 occurs via a similar pathway in the lung.

Taken together, our results indicate that up-regulation of miR-101 and/or miR144 could contribute to the suppression of CFTR observed in COPD patients. In addition, Clunes et al. recently showed that exposure of primary airway epithelial cells to short-term cigarette smoke lead to mucus dehydration [17]. Therefore, up-regulation of miR-101 by cigarette smoke or cadmium could affect lung fluid homeostasis and therefore mucus clearance by suppressing CFTR but also immune responses by preventing dephosphorylation of MAPKs due to inhibition of MKP-1. Future studies need to be done to investigate the effect of smoking cessation on CFTR expression and miRNAs regulating its expression. Our study highlights the role of miRNAs as genetic modifiers that may contribute to chronic bronchitis by altering expression of CFTR that regulates lung epithelial surface hydration.
